# The Disease Ontology: fostering interoperability between biological and clinical human disease-related data

**DOI:** 10.1007/s00335-015-9576-9

**Published:** 2015-06-21

**Authors:** Lynn M. Schriml, Elvira Mitraka

**Affiliations:** Institute for Genome Sciences, University of Maryland School of Medicine, Baltimore, MD 21201 USA

## Abstract

The Disease Ontology (DO) enables cross-domain data integration through a common standard of human disease terms and their etiological descriptions. Standardized disease descriptors that are integrated across mammalian genomic resources provide a human-readable, machine-interpretable, community-driven disease corpus that unifies the representation of human common and rare diseases. The DO is populated by consensus-driven disease data descriptors that incorporate disease terms utilized by genomic and genetic projects and resources engaged in studies to understand the genetics of human disease through the study of model organisms. The DO project serves multiple roles for the model organism community by providing: (1) a structured “backbone” of disease concepts represented among the model organism databases; (2) authoritative disease curation services to researchers and resource providers; and (3) development of subsets of the DO representative of human diseases annotated to animal models curated within the model organism databases.

## Introduction

The nomenclature of human disease has a long and complex history. Traditionally, diseases have been named for the discoverer of the disease (Robinow syndrome), where the disease was first discovered (Rocky Mountain spotted fever), or the animal in which the disease was first identified (avian influenza). A disease may initially be called a syndrome to describe a collection of symptoms. As genes, genetic variants and the etiology of a disease are discerned; the disease name may or may not be changed to reflect the new level of knowledge. Consequently, there is a significant need for a standardized representation of human diseases to map disease concepts across resources, to connect related information, and to support the development of computational tools that will enable robust data analysis and integration. Utilizing a standardized ontological representation of human diseases creates a rigorous knowledge backbone for the annotation of biomedical data through defined concepts connected by specified relations (Hoehndorf et al. 2012; Musen et al. 2012). The Disease Ontology (DO) (Schriml et al. 2012; Kibbe et al. 2015) was developed to connect clinical vocabulary concepts through the creation of a single unifying representation of human disease. The DO simplifies and facilitates the exchange and comparison of disease-related information between biomedical and clinical resources. The scope of this review is to highlight the roles of the DO project contributing to human disease and mammalian genetic research, specifically describing the DO’s activities to support mouse model organism research at the mouse genome informatics (MGI) database. The DO project provides on-going expert curation of human diseases annotated within the model organism databases (MOD) (Kibbe et al. 2015) and has begun to create MOD-specific subsets of DO to facilitate the use of DO within the MODs. The DO team has created a DO_MGI_slim as a subset of the DO for human disease terms associated with mouse animal models within MGI. A slim of the DO is a smaller version of the DO’s HumanDO.obo file that contains a subset of terms from the entire DO file.

## DO: a centralized disease term resource

The DO provides a homogeneous, centralized ontological representation of human disease that fosters precise identification and interpretation of disease data elements and incorporates the changing nomenclature of human disease terms over time along with community-specific naming conventions and synonyms.

The DO project was established in 2003 at Northwestern University to address the need for a purpose-built ontology that covers the full spectrum of disease concepts annotated within biomedical repositories within an ontological framework that is extensible to meet community needs. Disease concepts are defined for a variety of reporting and indexing purposes that are represented in numerous medical vocabularies. The DO was initially built from the union of disease terms extracted from the ICD mortality and morbidity classifications (Ayme et al. 2010), SNOMED-CT clinical and event healthcare reports (Donnelly 2006), and MeSH Medline index terms (Nelson et al. 2004; Kibbe et al. 2015). Expansion of disease sub-types has been driven by the disease data needs of the user community. The DO team works with several MODs to integrate their human disease terms into DO. The DO project additionally integrates human disease terms represented in biomedical resources including the Online Mendelian Inheritance in Man (OMIM) (Amberger et al. 2015), the experimental factor ontology (EFO) (Parkinson et al. 2011), the protein ontology (PRO) (Natale et al. 2014), and orphanet (Ayme et al. 2010) to provide authoritative and rigorously reviewed disease terms. DO’s inclusion of unique identifiers from the medical vocabularies facilitates cross-mapping between resources and enables the DO team to quickly identify and avoid inclusion of overlapping (not novel) content.

## DO development and content

As a community-driven standards-based ontology project, the DO is focused on representing common and rare disease concepts captured across biomedical resources with the mission of providing a broadly useful disease interface between data resources through on-going support (term review and integration) of disease terminology needs. The DO includes 6582 terms (38 % defined, revision 2821) representing inherited, developmental, and acquired human diseases. The HumanDO.obo file additionally includes the set of 2373 obsoleted DO terms to enable ID tracking. As terms are added or reviewed within the DO, textual definitions are added. The DO team is actively working to define all terms within DO. We have increased the percent of defined DO terms by 16 % since 2012. This includes the addition of 163 DO terms and an increase of defined DO terms by 6 % overall between October 2014 and May 2015. The DO team will continue to address the need to add definitions for our backlog of undefined terms.

## DO: representation of disease terms

DO disease descriptors represent individual disease terms and are provided in a computable format where each DO term is represented by unique uniform resource identifiers (URI’s) (e.g., http://www.purl.obolibrary.org/obo/DOID_4). A DOID is a disease ontology ID. The DO’s HumanDO.obo file can be downloaded from our Sourceforge repository at: http://www.sourceforge.net/p/diseaseontology/code/HEAD/tree/trunk/HumanDO.obo. The OWL version of the HumanDO file, produced by the OBO Foundry, can be found at: http://www.purl.obolibrary.org/obo/doid.owl. The DO project publishes our primary data files and software in public repositories to ensure that they will remain publicly available. While maintaining DO’s Sourceforge archive (http://www.sourceforge.net/p/diseaseontology) for current DO users, the DO project has began to transition our production activities to a GitHub repository (http://www.github.com/obophenotype/human-disease-ontology) in 2015. The DO project’s ontology and database files are freely available for copying, redistribution, and adaption for any purpose under the Creative Commons Attribution 3.0 Unported License (http://www.creativecommons.org/licenses/by/3.0/).

**Fig. 1:**
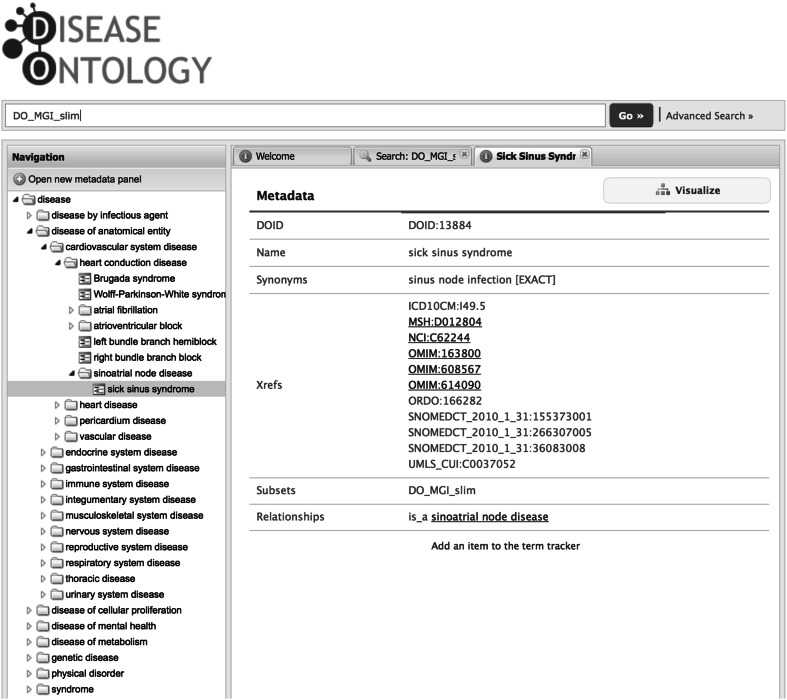
The disease ontology website. A query for all DO terms included in the DO_MGI_slim is displayed

The DO website (http://www.disease-ontology.org) contains a web-based application (Fig. 1) that enables full-text searching of DO disease terms, definitions, and related metadata. The DO includes a subset of terms utilized within MGI. The set of MGI human disease terms can be retrieved through the DO web interface by searching on the slim name DO_MGI_slim in the search box or as an Advanced Search by selecting subset. This query will retrieve the set of disease terms utilized for the annotation of human diseases for mouse animal models in MGI. Additionally, the set of DO diseases with an associated OMIM cross-reference can be retrieved by an Advanced Search of Xrefs and typing in OMIM to the query box.

The DO web browser’s RESTful API service enables direct querying of a single DOID (e.g., sick sinus syndrome http://www.disease-ontology.org/term/DOID:13884/) and programmatic access of the DO database metadata (http://www.disease-ontology.org/api/metadata/DOID:13884) to produce:

{“xrefs”: [“ICD10CM:I49.5”, “MSH:D012804”, “NCI:C62244”, “OMIM:163800”, “OMIM:608567”, “OMIM:614090”, “ORDO:166282”, “SNOMEDCT_2010_1_31:155373001”, “SNOMEDCT_2010_1_31:266307005”, “SNOMEDCT_2010_1_31:36083008”, “UMLS_CUI:C0037052”], “name”: “sick sinus syndrome”, “subsets”: [“DO_MGI_slim”], “synonyms”: [“sinus node infection EXACT “], “parents”: [[“is_a”, “sinoatrial node disease”, “DOID:0050824”]], “id”: “DOID:13884”}.

## DO: disease curation service and community collaborations

The DO project established a user support workflow in 2007 to invite DO users to submit DO term requests and updates. The DO team reviews, classifies, and integrates requested terms into DO. The DO team has established curation ‘best practices’ to field and respond to user requests, to coordinate disease representation within biomedical resources, and to provide subject matter and ontology expert curation guidance to the user community. DO curator’s Style Guide is available on the DO website under the Resources tab. The DO Style Guide provides documentation on defining new terms, cleaning up term format, creating definitions, dbxrefs (IDs as references to external resources), and provenience of term definition sources as URLs.

The DO project has established a network of collaborative disease annotation efforts where the community members contribute their expertise and area-specific disease content to DO and the DO team provides expert disease classifications to the community members (Kibbe et al. 2015). These on-going efforts ensure the accuracy of DO annotations through on-going community review and feedback provided to DO. Additionally, the DO team receives novel disease terms, suggestions, and advices from subject matter experts in an on-going weekly, sometimes daily, process. For example, the neuroscience information framework (NIF) (Imam et al. 2012) and NeuroDevNet (Portales-Casamar et al. 2013) have been major contributors to DO’s representation of neurological diseases. The DO project collaborates with a number of NIH-funded genomic resources including: MGI, Protein Ontology (PRO), the Immune Epitope Database (IEDB), Reactome, WormBase (Harris et al. 2014), FlyBase (Dos Santos et al. 2015), and the NHLBI LINCS project (Vempati et al. 2014). For example, the Reactome project (Croft et al. 2014) has a total of 1226 pathways and reactions (out of 120,965 total human reactions and pathways) that are annotated with a DO disease term, and 2167 proteins and complexes (out of 40,349 human proteins and complexes) annotated with one.

## MGI: Disease Ontology collaboration

The DO project teamed up with MGI in the fall of 2014 to provide review and integration of MGI’s genetic disease vocabulary terms (aligned to OMIM terms and IDs) into DO. The DO is being enriched by the novel content contributed by MGI representing genetic disease sub-types associated with new mouse models and recently reported human diseases. The MGI team generated and provided DO with a mapping file of 2606 disease terms with corresponding OMIM IDs. Manual review of each disease term and OMIM mapping identified a set of 1198 OMIM IDs that mapped to 260 existing DO terms and a set of 1408 OMIM IDs that had no corresponding DOIDs. These 1408 OMIM IDs were mapped to new DO terms. In total, 250 new DO terms were created, primarily representing syndromes and rare diseases. The DO term to OMIM ID mapping identified several one to many mappings between DOIDs and OMIM IDs. This work represents a substantial multi-month manual curation effort and highlights the importance of the manual reviews required to connect biological concepts. Automated mapping of disease terms between resources and vocabularies holds the promise of streamlining manual reviews (Rance et al. 2013). The DO project recognizes the potential of automated mapping of disease terms. However, the DO project has been cautious to integrate automated analysis into our workflow, as the precision (the proportion of true positives that are correctly identified as true positives) of these analyses is less than 100 %. This manual review process included: the reassessment of the hierarchy of each term; the re-evaluation of its definition, if one existed; the definition of the term, if one didn’t exist; addition of synonyms; and mapping of the term to other sources (ICD10 codes, Orphanet IDs) via cross-references. A small number of OMIM terms could not be included in DO as they represent a phenotype but not a disease such as the OMIM entries: “Plasmodium falciparum blood infection level” (OMIM: 248310); “Plasmodium falciparum quantitative trait locus 1” (OMIM:611384), and “susceptibility to migraine, with or without aura” (OMIM:610208).

## DO: an ontology and resource disease hub

The DO has been widely adopted and utilized as a standard representation of human disease in biomedical ontologies including the human phenotype ontology (HPO; Köhler et al. 2014), the infectious disease ontology (IDO; http://www.infectiousdiseaseontology.org), the influenza ontology, the NIFSTD ontology, the cell ontology (CL), the experimental factor ontology (EFO), the protein ontology (PRO), and the cardiovascular disease ontology (https://www.code.google.com/p/cvdo/). The DO has become a highly utilized disease data resource for evaluating and connecting diverse sets of data. DO terms are being utilized for identifying representative phenotype sets (Köhler et al. 2014; Schofield et al. 2010; Monarch Initiative http://www.monarchinitiative.org and HPO Phenomizer http://www.compbio.charite.de/phenomizer/) and functionally similar genes (Fang and Gough 2013; Singleton et al. 2014). The DO is also being used for analyzing ontologies and protein domain annotations (dcGOR (Fang 2014)), human gene and genome annotations (Peng et al. 2013; Osborne et al. 2009), pathways (Croft et al. 2014; Hoehndorf et al. 2012), cancer variants (Wu et al. 2014; Milacic et al. 2012), and immune epitopes (Vita et al. 2014).

The extensive use of DO in data analysis workflows and the annotation of DO terms across a wide body of genomic resources enables users to readily identify data of interest within or between these resources and to identify novel connections. For example, the Jackson Laboratory for Genomic Medicine is utilizing DO within their Clinical Genome Informatics System workflow to collect clinical disease data and to translate pathology reports to Next Generation Sequencing Clinical Reports (Ananda et al. 2015). Likewise, the BioXpress, a gene expression and cancer association database, is utilizing DO to map cancer terms across cancer resources to facilitate uniform pan-cancer analysis (Wan et al. 2015).

## DO subsets: resource-specific disease representations (DO slims)

By definition biomedical ontologies capture the breadth of domain specific knowledge. In order to take further advantage of the detailed ontological information found in the leaf ontology terms subsets of biomedical ontologies, called slims, are being created to provide customized high-level views (Davis et al. 2010; Geifman et al. 2010). For example, subsets of the gene ontology (GO) (GO Consortium 2013) have been created (http://www.geneontology.org/page/go-slim-and-subset-guide) to represent model organism or resource-specific subsets of GO. The DO project initiated the development of DO slims in 2014 to provide resource- (e.g., MGI) or disease-specific (e.g., cancer) subsets of DO. DO slim files can be extracted from the DO HumanDO.obo file and are also are available as from DO’s GitHub repository (https://www.github.com/obophenotype/human-disease-ontology).

The DO_MGI_slim represents a focused view of the subset of genetic diseases in DO that are represented in MGI and enables researchers to identify and explore mouse model organism associated diseases. The DO_MGI_slim subset currently contains 354 terms (DO revision 2821). Additional terms are being reviewed for inclusion in DO from a set of 510 MGI human disease terms. DO slim files are available from the DO SVN repository.

The DO_cancer_slim provides a broad view of the set of DO cancer terms represented within the catalog of somatic mutations in cancer (COSMIC), the international cancer genome consortium (ICGC), the cancer genome atlas (TCGA), and the integrative oncogenomics (IntOGen) database to support the coordinated effort of pan-genome analysis of cancer genetic variants (Wu et al. 2015). The DO_cancer_slim represents the classification of a subset of 393 cancer terms into a cohesive set of 192 DO terms. This collaborative classification of cancer terms resulted in 43 new cancer terms being added to DO and the addition of definitions and references for 63 DO parent terms and 187 DO child node (leaf) terms. The DO_cancer_slim terms were further categorized by the type of cancer they represent into a selection of 64 upper-level DO terms (TopNodes_DOcancerslim). This project has produced a unifying ontology of cancer terms that could be used to map data across various resources, representing a multitude of cancer types, and be utilized for pan-cancer analysis.

## Conclusions

The DO project is highly committed in our efforts to support genetic and genomic research as disease data and curation resource. As a centralized disease resource DO provides stable, long-term support of disease terms to be used by the MOD research community. The DO team provides reliable and responsive curation services to researchers and data providers. Requests for DO curation may be submitted through the DO website (http://www.disease-ontology.org), DO’s SVN term tracker (http://www.sourceforge.net/p/diseaseontology/feature-requests/), DO’s Contact Us web form, or by emailing the DO team members (email: DO PI - Lynn Schriml: lschriml@som.umaryland.edu).

The DO project will be creating additional DO slims for each of the MODs (e.g., Reactome, FlyBase, WormBase, RGD, SGD, and ZFIN) in the near future. These subsets of the DO will enable DO users to review the representation and classification of associated diseases, to compare the diseases represented between MODs and to compare the different animal models associated with a particular disease or types of diseases across species.
